# Ketogenic diet alleviates renal fibrosis in mice by enhancing fatty acid oxidation through the free fatty acid receptor 3 pathway

**DOI:** 10.3389/fnut.2023.1127845

**Published:** 2023-03-23

**Authors:** Yang Qiu, Xiaofan Hu, Cong Xu, Chenqi Lu, Rui Cao, Yanan Xie, Jun Yang

**Affiliations:** Institute of Organ Transplantation, Tongji Hospital, Tongji Medical College, Huazhong University of Science and Technology, Key Laboratory of Organ Transplantation, Ministry of Education, NHC Key Laboratory of Organ Transplantation, Key Laboratory of Organ Transplantation, Chinese Academy of Medical Sciences, Wuhan, China

**Keywords:** ketogenic diet, renal fibrosis, β-hydroxybutyrate, fatty acid oxidation, free fatty acid receptor 3

## Abstract

**Introduction:**

The ketogenic diet (KD), as a dietary intervention, has gained importance in the treatment of solid organ structural remodeling, but its role in renal fibrosis has not been explored.

**Methods:**

Male C57BL/6 mice were fed a normal diet or a KD for 6 weeks prior to unilateral ureteral obstruction (UUO), a well-established *in vivo* model of renal fibrosis in rodents. Seven days after UUO, serum and kidney samples were collected. Serum β-hydroxybutyrate (β-OHB) concentrations and renal fibrosis were assessed. NRK52E cells were treated with TGFβ1, a fibrosis-inducing cytokine, and with or without β-OHB, a ketone body metabolized by KD, to investigate the mechanism underlying renal fibrosis.

**Results:**

KD significantly enhanced serum β-OHB levels in mice. Histological analysis revealed that KD alleviated structural destruction and fibrosis in obstructed kidneys and reduced the expression of the fibrosis protein markers α-SMA, Col1a1, and Col3a1. Expression of the rate-limiting enzymes involved in fatty acid oxidation (FAO), Cpt1a and Acox1, significantly decreased after UUO and were upregulated by KD. However, the protective effect of KD was abolished by etomoxir (a Cpt1a inhibitor). Besides, our study observed that KD significantly suppressed UUO-induced macrophage infiltration and the expression of IL-6 in the obstructive kidneys. In NRK52E cells, fibrosis-related signaling was increased by TGFβ1 and reduced by β-OHB. β-OHB treatment restored the impaired expression of Cpt1a. The effect of β-OHB was blocked by siRNA targeting free fatty acid receptor 3 (FFAR3), suggesting that β-OHB might function through the FFAR3-dependent pathway.

**Discussion:**

Our results highlight that KD attenuates UUO-induced renal fibrosis by enhancing FAO via the FFAR3-dependent pathway, which provides a promising dietary therapy for renal fibrosis.

## Introduction

Over 10% of the world's population are suffering from chronic kidney disease (CKD) (1). As this disease progresses, CKD eventually develops into end-stage renal disease, requiring that patients receive dialysis or kidney transplantation. Renal fibrosis is a hallmark and common outcome in all types of progressive CKD, including chronic allograft nephropathy. Renal fibrosis is characterized by inflammation, myofibroblast activation and migration, excess deposition of the extracellular matrix, and renal structural remodeling. Its typical pathological presentations indicate interstitial fibrosis and tubular atrophy (2–4). Because the intricate mechanisms of renal fibrosis remain unclear, there is still a lack of feasible targeted therapies that can alleviate or reverse fibrosis progression.

Recent studies report that insufficient energy supply from fatty acid oxidation (FAO) in cardiac myocytes and tubular epithelial cells is an important mechanism of myocardial/renal dysfunction or failure (5–8). With respect to what is known about this catabolic pathway, carnitine palmitoyltransferase 1a (Cpt1a) and acyl-coenzyme A oxidase 1 (Acox1) are its rate-limiting enzymes in FAO. Cluster of differentiation 36 (CD36) facilitates the uptake of long-chain fatty acids (9), and peroxisome proliferator-activated receptor-α (PPARα) and PPAR-γ coactivator-1a (PPARGC1a) are the key transcription factors that regulate the expression of target genes (Cpt1a and Acox1) involved in FAO (10, 11). Studies have found that ketogenic diet (KD) enhances myocardial FAO to prevent cardiac dysfunction and fibrosis in mice (5, 6).

Dietary intervention has become one of the most important non-drug therapies, especially for metabolic diseases. KD has been used as an approach to treat drug-resistant epilepsy for over 70 years (12). Recently, KD has generated a lot of interest due to its beneficial impact in various diseases, including Alzheimer's disease, obesity, cardiovascular diseases, cancer, and diabetes (13). KD is a high-fat, extremely low-glucose diet that enhances the metabolism of ketone bodies, including acetoacetate, β-hydroxybutyrate (β-OHB), and acetone. KD is considered a potential dietary intervention to treat solid organ structural remodeling, but its role in renal fibrosis has not been explored. Therefore, we examined the effects of KD on renal fibrosis induced by unilateral ureteral obstruction (UUO) in mice and elucidated the underlying mechanisms, which provides an acceptable dietary regimen for renal fibrosis.

## Materials and methods

### Animals

All procedures conformed to the Chinese Council on Animal Care guidelines. Our study was approved by the Institutional Animal Care and Use Committee of Tongji Medical College of Huazhong University of Science and Technology (Approval Number: TJH-202111009). Male C57BL/6 mice (8 weeks old, weighing 18–20 g) were purchased from Weitonglihua Laboratory Animal Technology (Beijing, China) and maintained under constant environmental and specific pathogen-free conditions. A portion of the C57BL/6 mice in this experiment underwent a UUO operation, a well-established *in vivo* model of disease progression in rodents, as previously reported (14). Briefly, UUO was conducted by double-knot ligation of the middle and upper segments of the left ureter after the mice were anesthetized. Serum and left kidney samples were obtained 7 d after surgery in the non-fasted mice. C57BL/6 mice were randomly divided into four groups: those fed a normal diet (“normal”), those fed KD (“KD sham”), those fed a normal diet who had undergone the UUO operation (“ND+UUO”), and those fed KD those who had undergone the UUO operation (“KD+UUO”). Six mice were used in each group. All mice were fed normal diet or KD ad libitum for 6 weeks prior to UUO. KD was purchased from Weitonglihua Laboratory Animal Technology and consisted of nearly 90% calories from fat and 10% calories from protein. Etomoxir (MCE, Shanghai, China, 60 mg/kg body weight for 6 d) was injected intraperitoneally 1 d before UUO.

### Cell culture

NRK52E cells (rat kidney tubular epithelial cells [TECs]) were used *in vitro* and cultured in the Dulbecco's modified Eagle's medium (DMEM) (10% fetal bovine serum, Gibco, Invitrogen, Carlsbad, CA, USA) at 37°C in 5% CO2. The cells were digested with trypsin and seeded in six-well plates as required. After growing for 24 h, the cells were treated with recombinant transforming growth factor β1 (TGFβ1, 10 ng/mL, PeproTech, Rocky Hill, NJ, USA) with or without β-OHB (10 mM, MCE) for 48 h. A free fatty acid receptor 3 (FFAR3) agonist, AR420626 (5 μM; MCE), was used to verify the effect of FFAR3. Cells were co-stimulated with AR420626 and TGFβ1 for 48 h.

### Gene knockdown of FFAR3 by small interfering RNA

Transfection with siRNA against rat FFAR3 (Target RefSeqID: NM_001108912.1, *FFAR3* gene of the Rattus norvegicus species; Sequences: Sence-GGAAUGUCCGAGCUAGAGATT; Antisense-UCUCUAGCUCGGACAUUCCTT) was purchased from AuGCT Biotechnology Company, Wuhan, China. NRK52E cells were transfected with negative control siRNA or siRNA targeting FFAR3 using Lipo6000 Transfection Reagent (Beyotime, Shanghai, China) 24 h before TGFβ1 stimulation. After 24 h of incubation, cells were treated with TGFβ1 and with or without β-OHB.

### Measurement of serum β-OHB

The concentration of serum β-OHB 7 d after UUO was detected using a β-OHB detection kit (Jiancheng Bioengineering Institute, Nanjing, China) according to the manufacturer's instructions.

### Histologic analysis and immunohistochemistry

Paraffin-embedded left kidney tissue sections were stained with hematoxylin and eosin (H&E) to evaluate the severity of glomerular and tubular interstitial injury 7 d after UUO. Masson's trichrome and picrosirius red staining were performed to measure interstitial fibrosis in the kidneys. Fluorescence microscopic examination (Nikon Eclipse, Tokyo, Japan) showed that collagen fibers were stained blue in Masson's trichrome staining and red in picrosirius red-stained kidney tissues. Fibrosis area calculation was conducted in a blinded manner using at least five randomly selected fields from each kidney section using Image-Pro Plus, version 6.0.

IHC was performed on paraffin-embedded kidney sections. Primary antibodies against α-smooth muscle actin (anti-α-SMA, 1:1000, Abcam, Shanghai, China), anti-collagen type I alpha 1 chain (anti-Col1a1, 1:800, Abcam), and anti-F4/80 (1:500, Cell Signaling Technology, Danvers, MA, USA) were used. The positive area of immunohistochemical staining was calculated using 10 randomly selected fields at × 400 magnification with Image-Pro Plus, version 6.0.

### Immunofluorescence

For immunofluorescence analysis of NRK52E cells, cells on glass coverslips were fixed with ice-cold methanol for 10 min and then permeabilized with 0.1% Triton for 5 min at 25°C. The cells were blocked with 5% bovine serum albumin (BSA, Biosharp, Beijing, China), incubated overnight at 4°C with primary antibodies (anti-Cpt1a, 1:100, Abclonal, Wuhan, China), and then incubated in the dark with secondary antibodies (DyLight 488 goat anti-rabbit IgG, 1:500, Abbkine, Wuhan, China) for 1 h at 25°C. Finally, nuclei were stained with Hoechst (Beyotime) for 5 min.

Immunofluorescence analysis of the left kidney tissue was performed on paraffin-embedded sections mounted on glass slides. Primary antibody anti-F4/80 (1:1000, Cell Signaling Technology) and HRP-goat anti-rabbit IgG secondary antibody (1:4000, Abcam) were used. The slides were visualized under a fluorescence microscope (Nikon Eclipse, Tokyo, Japan).

### Western blot analysis

Proteins from kidney tissues and cultured cells were extracted with RIPA lysis buffer (Beyotime). Protease and phosphatase inhibitor cocktails were added for the extraction of phosphorylated proteins. The total protein concentration was measured using a BCA protein assay kit (Beyotime). Proteins (40–80 μg) were separated using SDS-PAGE (10%) and transferred to a PVDF membrane. The membranes were blocked with 5% BSA for 1.5 h and incubated with primary antibodies overnight at 4°C, followed by secondary antibodies (goat anti-rabbit, 1:3000; anti-mouse, 1:5000; Servicebio, Wuhan, China) for 1.5 h. The following primary antibodies were used: anti-α-SMA (1:3000, Proteintech, Wuhan, China), anti-Col1a1 (1:1000, Cell Signaling Technology), anti-collagen type III alpha 1 chain (anti-Col3a1, 1:1000, Abclonal), anti-Cpt1a (1:1000, Abclonal), anti-Acox1 (1:1000, Abclonal), anti-phosphorylation-AMP-activated protein kinase (anti-p-AMPK, 1:1000, Abclonal), and anti-glyceraldehyde-phosphate dehydrogenase (anti-GAPDH, 1:50000, Abclonal). Images were visualized using a Gene Gnome XRQ system (Syngene, Cambridge, UK).

### Real-time quantitative polymerase chain reaction

Total RNA was isolated from kidney tissues and cultured cells using an RNAfast200 reagent kit (Fastagen Biotech, Shanghai, China) and reverse-transcribed using a cDNA synthesis reagent kit (Yeasen, Wuhan, China). RT-qPCR was conducted using SYBR Green qPCR Master Mix (Vazyme, Wuhan, China). The primers used to amplify the specific gene fragments are listed in Supplementary Table S1. All samples were normalized to the housekeeping gene GAPDH and analyzed in triplicate using the ΔΔCT value method in StepOne Software v2.3.

### Statistical analysis

All data are presented as mean ± standard error of the mean (SEM). Three or more group comparisons were made using one-way analysis of variance (ANOVA) followed by Tukey's post-hoc tests in GraphPad Prism 9. Statistical significance was set at *p* < 0.05.

## Results

### KD significantly ameliorated renal fibrosis in the UUO mouse model

**Figure 1:**
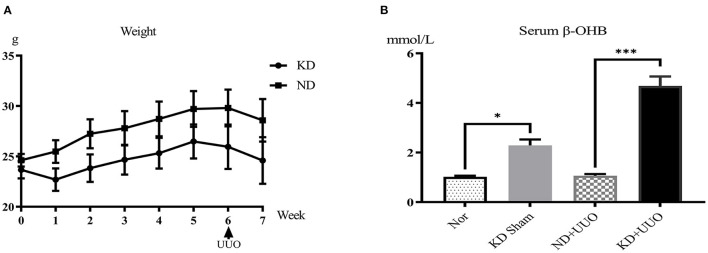
Ketogenic diet slowed down weight gain in mice and enhanced the β-OHB level in the serum. **(A)** The weight change of mice fed with ND or KD within 6 weeks before UUO and 1 week after UUO. **(B)** The serum β-OHB level (mmol/L) 7 d after UUO in all groups. Data are expressed as mean ± SEM. ***P < 0.001, *P < 0.05 as determined by one-way analysis of variance (ANOVA) followed by Tukey's post-hoc tests. Data shown are representative of 3 replicate experiments. KD, ketogenic diet; ND, normal diet; UUO, unilateral ureteral obstruction; β-OHB, β-hydroxybutyrate.

**Figure 2:**
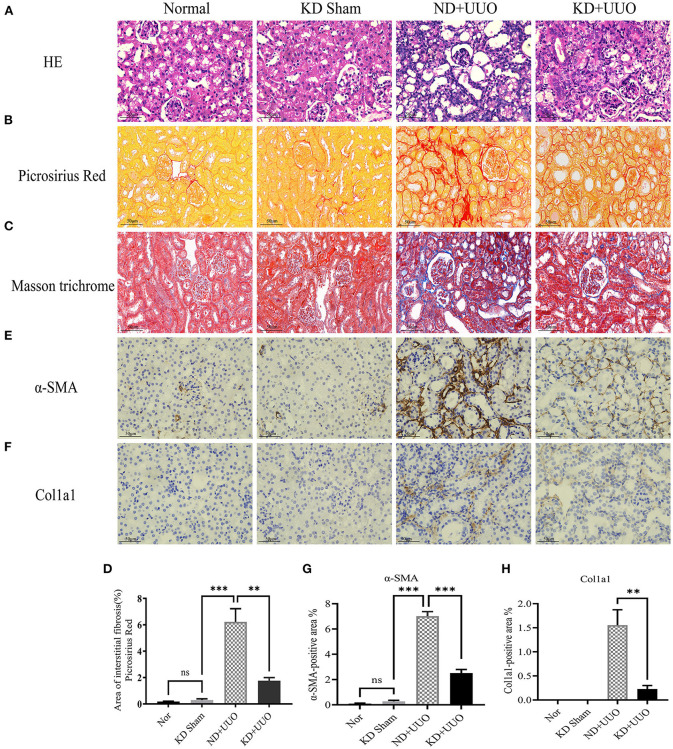
Ketogenic diet significantly alleviated structural destruction and excessive extracellular matrix deposition caused by UUO. **(A)** Histopathological examination (H&E) of kidney tissues in all groups showing structural destruction 7 d after UUO. **(B**–**D)** Masson's trichrome and picrosirius red staining of kidney sections and quantitative analysis of picrosirius red-stained sections. **(E, G)** Immunohistochemical staining images and quantitative analysis showing positive area of α-SMA 7 d after UUO in left kidney tissues from all groups. **(F, H)** Immunohistochemical staining images and quantitative analysis showing positive area of Col1a1 in kidney sections. Data are expressed as mean ± SEM. ***P < 0.001; **P < 0.01; ns, no significance as determined by one-way analysis of variance (ANOVA) followed by Tukey's post-hoc tests. Data shown are representative of 3 replicate experiments. KD, ketogenic diet; ND, normal diet; UUO, unilateral ureteral obstruction; α-SMA, α-smooth muscle actin; Col1a1, collagen type I alpha 1 chain.

**Figure 3:**
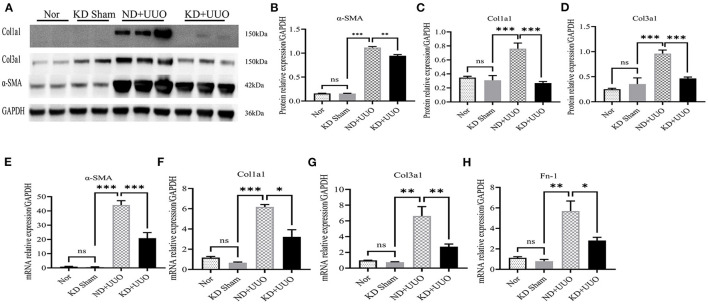
Ketogenic diet treatment alleviated UUO-induced renal fibrosis at the protein and mRNA expression level. **(A)** Western blotting analysis indicating the expression level of α-SMA, Col1a1 and Col3a1 7 d after UUO in kidney tissues from all groups. **(B–D)** Quantification of western blotting bands normalized to proteins bands of GAPDH. **(E–H)** Real-time quantitative polymerasechain reaction (RT-qPCR) indicating the mRNA expression level of α-SMA, Col1a1, Col3a1, and Fn-1 7 d after UUO in kidney tissue samples in the indicated groups. Data are expressed as mean ± SEM. ***P < 0.001; **P < 0.01; *P < 0.05; ns, no significance as determined by one-way analysis of variance (ANOVA) followed by Tukey's post-hoc tests. Data shown are representative of 3 replicate experiments. KD, ketogenic diet; ND, normal diet; UUO, unilateral ureteral obstruction; α-SMA, α-smooth muscle actin; Col1a1, collagen type I alpha 1 chain; Col3a1, collagen type III alpha 1 chain; GAPDH, glyceraldehyde-3-phosphate dehydrogenase; Fn-1, fibronectin-1.

To investigate the effects of KD on kidney fibrosis, we fed mice either a KD or a normal diet for 6 weeks and monitored weekly weight changes in mice. The weight gain of mice fed the KD was slower than that of mice fed a normalv diet (Figure 1A). KD significantly enhanced β-OHB levels in the serum of mice (ND+UUO vs. KD+UUO: 1.068 ± 0.063 mol/L vs. 4.689 ± 0.377 mmol/L, *P* < 0.001, Figure 1B). H&E staining revealed that structural destruction caused by ureteral obstruction was significantly alleviated by KD (Figure 2A). Masson's trichrome and picrosirius red staining demonstrated that KD effectively attenuated excessive collagen deposition in the interstitium of obstructed kidneys (Figures 2B, C). Quantification of picrosirius red staining showed an apparent decrease in the fibrotic area in the obstructed kidneys from KD feeding mice compared to those in the ND+UUO group (Figure 2D, from 6.226 ± 0.996% to 1.765 ± 0.229%). Using IHC and western blotting, we analyzed the protein markers of fibrosis, including α-SMA, Col1a1, and Col3a1, and further confirmed that the obstructed kidneys at 7 d after UUO were protected from the development of apparent fibrosis through KD. IHC showed that the positive areas of α-SMA and Col1a1 were drastically reduced in the KD+UUO group compared to the ND+UUO group (α-SMA: from 7.015 ± 0.362% to 2.514 ± 0.285%, *P* < 0.001; Col1a1: from 1.555 ± 0.319% to 0.230 ± 0.071%, *P* < 0.01; Figures 2E–H). Western blotting revealed that the protein expression levels of α-SMA, Col1a1, and Col3a1 were significantly upregulated by UUO (*P* < 0.001, Figures 3A–D). Upon intervention with KD, this increased fibrotic expression was attenuated (α-SMA, *P* < 0.01; Col1a1, *P* < 0.001; Col3a1, *P* < 0.001; Figures 3A–D). In accordance with protein expression, the mRNA expression levels of α*-SMA, Col1a1, Col3a1*, and fibronectin-1 (*Fn-1*) were attenuated in obstructed kidneys from the KD+UUO group, in contrast to those in the ND+UUO group (α-SMA, *P* < 0.001; Col3a1, *P* < 0.01; Col1a1 and Fn-1, *P* < 0.05; Figures 3E–H). These results suggested that KD treatment alleviated UUO-induced renal fibrosis *in vivo*.

### KD enhanced FAO and mitigated renal fibrosis

**Figure 4:**
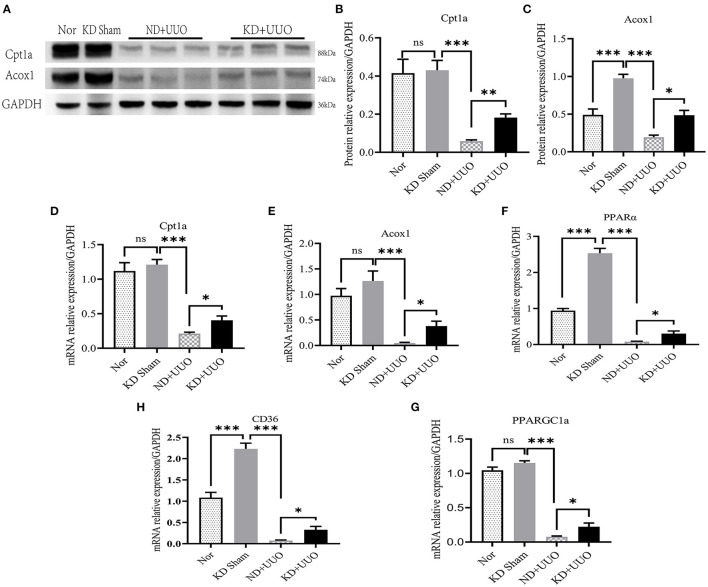
Ketogenic diet significantly restored the impaired expression levels of Cpt1a and Acox1 caused by UUO and enhanced the FAO pathway *in vivo*. **(A)** Western blotting analysis indicating the protein expression level of Cpt1a and Acox1 7 d after UUO in kidney tissues of mice. **(B, C)** Quantification of western blotting bands normalized to proteins bands of GAPDH. **(D–H)** Real-time quantitative polymerasechain reaction (RT-qPCR) indicating the mRNA expression level of Cpt1a, Acox1, PPARα, PPARGC1a and CD36 7 d after UUO in kidney tissues. Data are expressed as mean ± SEM. ***P < 0.001; **P < 0.01; *P < 0.05; ns, no significance as determined by one-way analysis of variance (ANOVA) followed by Tukey's post-hoc tests. Data shown are representative of 3 replicate experiments. KD, ketogenic diet; ND, normal diet; UUO, unilateral ureteral obstruction; Cpt1a, carnitine palmitoyltransferase 1a; Acox1, acyl-coenzyme A oxidase 1; GAPDH, glyceraldehyde-3-phosphate dehydrogenase; PPARα, peroxisome proliferator-activated receptor-α; PPARGC1a, PPAR-γ coactivator-1a; CD36, cluster of differentiation 36.

Recent studies have shown that impaired FAO plays a vital role in the development of renal fibrosis (7, 8, 15). Therefore, we hypothesized that KD improved renal fibrosis by enhancing the activity of FAO pathway. First, we checked the expression levels of the rate-limiting enzymes in FAO, Cpt1a, and Acox1. Significantly decreased protein expression of Cpt1a and Acox1 was observed in the obstructed kidneys 7 d after UUO in mice fed with ND, compared to that in normal kidneys (*P* < 0.001, Figures 4A–C). KD upregulated the protein levels of Cpt1a and Acox1 and enhanced FAO pathway activity (Cpt1a, *P* < 0.01; Acox1, *P* < 0.05; Figures 4A–C). Changes in the expression of these proteins were further verified by RT-qPCR (Figures 4D, E). Next, we examined other genes involved in fatty acid uptake, oxidation, and synthesis. Consistent with the changes in Cpt1a and Acox1, the mRNA expression levels of *PPAR*α*, PPARGC1a*, and *CD36* were dramatically reduced in obstructed kidneys (*P* < 0.001) and partially restored by KD (*P* < 0.05, Figures 4F–H).

**Figure 5:**
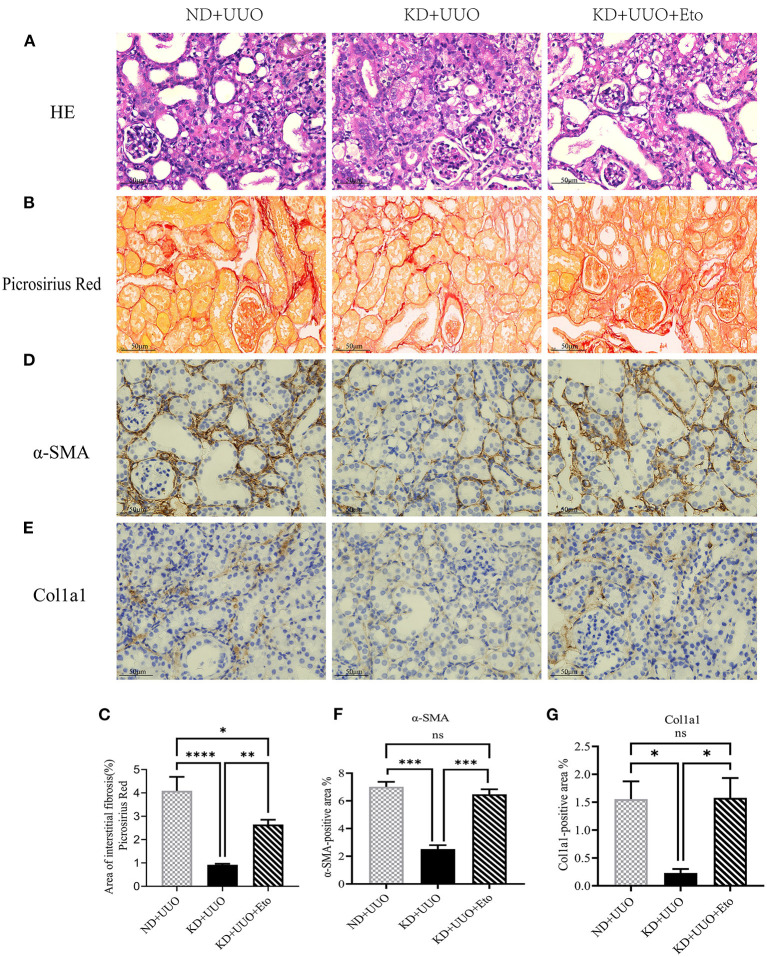
The protective effect of ketogenic diet on renal structural destruction and excessive extracellular matrix deposition was abolished by etomoxir. **(A)** Histopathological examination (H&E) of kidney tissues in all groups showing more severe tubular damage 7 d after UUO in the mice treated with etomoxir. **(B, C)** Picrosirius red staining of kidney sections and quantitative analysis of picrosirius red-stained sections. **(D, F)** Immunohistochemical staining images and quantitative analysis showing positive area of α-SMA 7 d after UUO in kidney tissues. **(E, G)** Immunohistochemical staining images and quantitative analysis showing positive area of Col1a1 7 d after UUO in kidney sections. Data are expressed as mean ± SEM. ***P < 0.001; **P < 0.01; *P < 0.05; ns, no significance as determined by one-way analysis of variance (ANOVA) followed by Tukey's post-hoc tests. Data shown are representative of 3 replicate experiments. KD, ketogenic diet; ND, normal diet; UUO, unilateral ureteral obstruction; Eto, etomoxir; α-SMA, α-smooth muscle actin; Col1a1, collagen type I alpha 1 chain.

**Figure 6:**
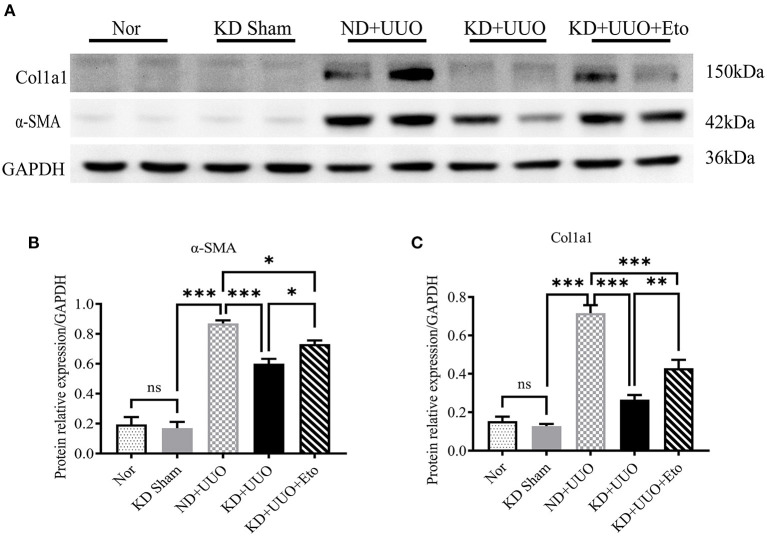
Etomoxir inhibited the FAO pathway and aggravated renal fibrosis 7 d after UUO. **(A)** Western blotting analysis indicating the protein expression level of α-SMA and Col1a1in kidney tissues in all groups. **(B, C)** Quantification of western blotting bands normalized to proteins bands of GAPDH. Data are expressed as mean ± SEM. ***P < 0.001; **P < 0.01; *P < 0.05; ns, no significance as determined by one-way analysis of variance (ANOVA) followed by Tukey's post-hoc tests. Data shown are representative of 3 replicate experiments. KD, ketogenic diet; ND, normal diet; UUO, unilateral ureteral obstruction; Eto, etomoxir; α-SMA, α-smooth muscle actin; Col1a1, collagen type I alpha 1 chain; GAPDH, glyceralde-hyde-3-phosphate dehydrogenase.

To further explore the role of FAO in kidney fibrosis, we used a Cpt1a inhibitor, etomoxir, in the UUO mouse model. Picrosirius red and H&E staining showed more collagen deposition and more severe tubular damage 7 d after UUO in the obstructed kidneys of mice treated with etomoxir and KD, compared with those fed with KD alone (Area of fibrosis, ND+UUO vs. KD+UUO vs. KD+UUO+Eto: 4.088 ± 0.599% vs. 0.924 ± 0.041% vs. 2.650 ± 0.201%, *P* < 0.01; Figures 5A–C). IHC analyses revealed that the α-SMA-positive and Col1a1-positive areas significantly increased with etomoxir treatment in the kidneys (ND+UUO vs. KD+UUO vs. KD+UUO+Eto, α-SMA: 7.015 ± 0.362% vs. 2.514 ± 0.285% vs. 6.476 ± 0.364%, *P* < 0.001; Col1a1: 1.555 ± 0.319% vs. 0.230 ± 0.071% vs. 1.580 ± 0.353%, *P* < 0.05; Figures 5D–G). These changes were further determined based on the expression of fibrosis-associated proteins. Compared with the kidneys from the mice fed with KD alone, the obstructed kidneys treated with both etomoxir and KD showed higher expression levels of α-SMA and Col1a1 7 d after UUO, but still less than those from the mice in the ND+UUO group (α-SMA, ND+UUO vs. KD+UUO: *P* < 0.001, KD+UUO vs. KD+UUO+Eto: *P* < 0.05; Col1a1, ND+UUO vs. KD+UUO: *P* < 0.001, KD+UUO vs. KD+UUO+Eto: *P* < 0.01; Figures 6A–C). Collectively, the obstructed kidneys treated with etomoxir suffered more severe renal injuries and fibrosis, although these mice were fed KD. The effect of KD on renal fibrosis was attenuated when FAO was inhibited. In brief, these data indicated that FAO played an important role in renal fibrosis, and we concluded that KD might effectively alleviate renal fibrosis induced by UUO through improving FAO.

### KD reduced macrophage infiltration in the UUO mouse model

**Figure 7:**
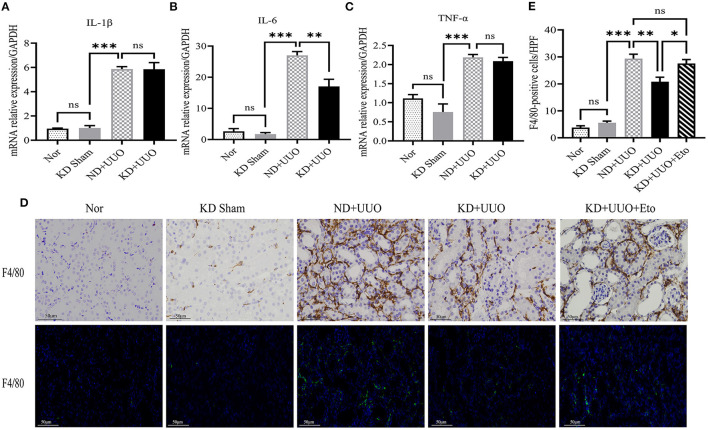
Ketogenic diet reduced the release of the proinflammatory cytokine and macrophage infiltration in the obstructed kidney. **(A–C)** Real-time quantitative polymerasechain reaction (RT-qPCR) indicating the mRNA expression level of IL-1β, IL-6 and TNF-α 7 d after UUO in kidney tissues. **(D)** Immunohistochemical and immunofluorescence images of stained kidney sections with F4/80-specific antibody indicating the number of macrophages 7 d after UUO. **(E)** Quantitative analysis of the number of F4/80-positive cells/HPF. Data are expressed as mean ± SEM. ***P < 0.001; **P < 0.01; *P < 0.05; ns, no significance as determined by one-way analysis of variance (ANOVA) followed by Tukey's post-hoc tests. Data shown are representative of 3 replicate experiments. KD, ketogenic diet; ND, normal diet; UUO, unilateral ureteral obstruction; Eto, etomoxir; GAPDH, glyceraldehyde-3-phosphate dehydrogenase; IL, interleukin; TNF-α, tumor necrosis factor-α; HPF, high power field.

Inflammation is involved in the progression of fibrosis and plays an important role in tissue damage and repair (16). Previous studies have shown that macrophages contribute to the development of fibrosis (16–18). We further detected the expression of inflammatory indices in obstructed kidneys. The UUO-induced pro-inflammatory cytokines/chemokines, such as interleukin-1β (*IL-1*β), interleukin-6 (*IL-6*), and tumor necrosis factor-α (*TNF-*α), were increased 7 d after UUO (*P* < 0.001, Figures 7A–C). The expression of *IL-6* was downregulated in the kidneys of the KD + UUO group (*P* < 0.01, Figure 7B). However, our data demonstrated no significant reduction in the expression of *IL-1*β and *TNF-*α in the KD+UUO group (Figures 7A, C). In our study, KD significantly suppressed UUO-induced infiltration of macrophages identified by the surface marker F4/80 in obstructed kidneys (ND+UUO vs. KD+UUO: 29.38 ± 1.625 vs. 20.83 ± 1.621 cells/HPF, *P* < 0.01; Figures 7D, E). However, this protective effect was blocked by etomoxir (Figure 7E).

### β-OHB enhanced FAO activity and improved fibrosis *via* the FFAR3-dependent pathway in TECs

Next, we explored the mechanism of renal fibrosis improvement by KD in renal TECs. β-OHB is not only a simple energy intermediate metabolite but also plays an important role as a signaling molecule in different physiological contexts (19, 20). NRK52E cells were used to examine how β-OHB, as the most abundant form of ketone bodies, modified FAO pathway activity.

**Figure 8:**
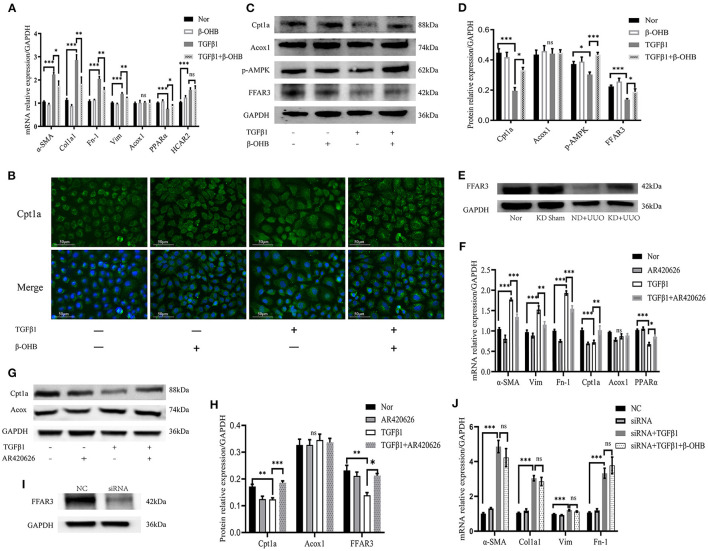
β-OHB alleviated fibrosis by enhancing FAO via the FFAR3-dependent pathway in NRK52E. **(A)** Real-time quantitative polymerasechain reaction (RT-qPCR) indicating the mRNA expression level of α-SMA, Col1a1, Fn-1, Vim, Acox1, PPARα and HCAR2 in TECs. After incubation for 24h, the cells were treated with TGFβ1 (10 ng/mL) with or without β-OHB (10 mM) for 48h. **(B)** Immunofluorescence images of cultured cells indicating cellular localization of Cpt1a (green). The nuclear region was stained with Hoechst (blue). Cell culture and stimulation was performed as above. **(C, D)** Quantification analysis of western blotting indicating the protein expression level of Cpt1a, Acox1, p-AMPK and FFAR3 in cultured cells. **(E)** Western blotting analysis indicating the protein expression level of FFAR3 7 d after UUO in kidney tissues of mice. **(F)** RT-qPCR showing the mRNA expression level of α-SMA, Fn-1, Vim, Cpt1a, Acox1, and PPARα in TECs. The cells were co-stimulated with TGFβ1 (10 ng/mL) and AR420626 (5 μM) for 48h. **(G, H)** Quantification analysis of western blotting indicating that AR420626 reverted significantly the inhibition of Cpt1a and FFAR3 induced by TGFβ1 in NRK52E cells. **(I)** Western blotting analysis indicating the decreased protein expression level of FFAR3 by siRNA. **(J)** The mRNA expression level of α-SMA, Col1a1, Fn-1, and Vim in cultured cells determined by RT-qPCR. The NRK52E cells were transfected with control siRNA or siRNA against FFAR3 at 24h before TGFβ1 stimulation. After 24h incubation, the cells were further treated with TGFβ1 with or without β-OHB. Data are expressed as mean ± SEM. ***P < 0.001; **P < 0.01; *P < 0.05; ns, no significance as determined by one-way analysis of variance (ANOVA) followed by Tukey's post-hoc tests. Data shown are representative of 3 replicate experiments. β-OHB, β-hydroxybutyrate; FAO, fatty acid oxidation; TECs, kidney tubular epithelium cells; TGFβ1, transforming growth factor β1; GAPDH, glyceraldehyde-3-phosphate dehydrogenase; α-SMA, α-smooth muscle actin; Col1a1, collagen type I alpha 1 chain; Fn-1, fibronectin-1; Vim, vimentin; Acox1, acyl-coenzyme A oxidase 1; PPARα, peroxisome proliferator-activated receptor-α; HCAR2, hydroxycarboxylic acid receptor 2; Cpt1a, carnitine palmitoyltransferase 1a; p-AMPK, phosphorylation-AMP-activated protein kinase; FFAR3, free fatty acid receptor 3; KD, ketogenic diet; ND, normal diet; UUO, unilateral ureteral obstruction; NC, negative control; siRNA, small interfering RNA.

The mRNA expression levels of fibrosis-related signaling molecules, including α*-SMA, Col1a1, Fn-1*, and vimentin (*Vim*), were elevated by TGFβ1 and markedly downregulated by β-OHB after stimulation for 48 h (*P* < 0.01, Figure 8A). Next, we measured FAO-associated gene expression and determined that Cpt1a was significantly expressed in the cytoplasm of rat TECs (Figure 8B). Treatment with β-OHB for 48 h restored the impaired expression of Cpt1a and *PPAR*α, consistent with our *in vivo* experiments (*P* < 0.05, Figures 8A–D). In addition, β-OHB is known to activate AMPK to ameliorate inflammasomes (21). We observed remarkably increased phosphorylation of AMPK by β-OHB (*P* < 0.001, Figures 8C, D). We then investigated the binding mechanism of β-OHB to the cell surface. β-OHB is currently known to bind to two classes of G-protein-coupled receptors: hydroxycarboxylic acid receptor 2 (HCAR2) and FFAR3 (19, 20). We detected changes in the expression of the two receptors using PCR and western blotting. Although the mRNA level of *HCAR2* was markedly elevated by TGFβ1 (*P* < 0.001), β-OHB did not alter its expression in TECs (Figure 8A). Conversely, β-OHB significantly reverted the reduced expression of FFAR3 induced by TGFβ1 (*P* < 0.05, Figures 8C, D). Consistent with the changes *in vitro*, the protein expression level of FFAR3 dramatically decreased in obstructed kidneys and was restored by KD in mice (Figure 8E).

To further demonstrate the role of FFAR3 in fibrosis, a FFAR3 agonist, AR420626, was used to mimic the β-OHB activation pathway. Similarly, AR420626 significantly mitigated fibrosis induced by TGFβ1 and greatly increased FAO after co-stimulation with TGFβ1 for 48 h (Figures 8F–H), suggesting that activation of FFAR3 might increase the FAO activity and eventually mitigate fibrosis. Next, we knocked down the expression of FFAR3 using siRNA to examine whether β-OHB functioned through the FFAR3-dependent pathway in TECs. The expression of FFAR3 was inhibited immensely by siRNA (Figure 8I). The protective effect of β-OHB on fibrosis was abolished by siRNA in TECs (Figure 8J). In summary, our results indicated that β-OHB mitigated fibrosis by enhancing cellular FAO via the FFAR3-dependent pathway.

## Discussion

In this study, we observed for the first time that KD attenuates UUO-induced renal fibrosis and protects renal structure from obstructive destruction *in vivo*. Moreover, KD significantly reduces macrophage infiltration in renal tissue. The effects of KD on renal fibrosis depend mainly on its enhancement of FAO activity. Additionally, our work *in vitro* revealed that β-OHB enhances FAO *via* FFAR3, which improves fibrosis in TECs. These findings demonstrate that KD represents a promising dietary therapeutic strategy for treating renal fibrosis and CKD.

The UUO in rodents is conducted to mimic human obstructive nephropathy. The UUO-induced renal fibrosis is characterized by severe structural damage, including a decreased number of tubules, tubular atrophy, dilated tubules, and excess deposition of the extracellular matrix, eventually leading to the common pathological manifestations of kidney fibrosis (14). In recent years, deficient FAO in renal TECs has been found to be crucial for renal fibrosis (7, 8, 15, 22). As highly metabolic cells, TECs prefer FAO and maintain high expression level of FAO- related enzymes, which generates more energy than the oxidation of glucose (7, 8, 15, 23). Impairment of FAO in TECs induces ATP depletion, intracellular lipid deposition, cell death, and dedifferentiation, eventually leading to fibrosis (7, 8). Our results also confirmed severely impaired FAO in the UUO-induced fibrosis model. Previous studies (7, 8, 15) and our results indicate that restoration of impaired FAO effectively prevents the progression of renal fibrosis. Thus, targeted therapies for FAO in the early stages of fibrosis provide new insights into the prevention of renal fibrosis.

Dietary interventions for the treatment of various diseases are drawing widespread attention and have gained increasing importance. KD, as a typical dietary intervention, is characterized by the supply of a large mass of calories from fat, a small proportion derived from protein, and very few calories from glucose. In the context of constant total calorie intake, KD enhances ketogenesis, suppresses oxidative stress, increases insulin sensitivity, and significantly increases FAO in the body (13, 24–26). Two recent heart studies have found that KD reversed cardiac fibrosis by enhancing myocardial FAO metabolism in mice (5, 6). Similar results were found in our study on the kidneys. In this study, we clearly indicated that KD promoted the absorption and oxidation of fatty acids, improved energy metabolism dysfunction, and protected against renal fibrosis in the UUO-induced fibrosis model. KD alone could not significantly enhance FAO in the sham mice consuming KD probably because of the relatively high level of FAO in normal kidneys. In addition, KD alleviates pulmonary fibrosis and effectively slows polycystic kidney disease progression through the inhibition of mTOR signaling (27, 28). However, another study reports that KD inhibits mitochondrial biogenesis and promotes cardiac fibrosis (29). The reason for this difference might lie in the animal model, highlighting the detrimental effects of long-term KD in normal rats. Furthermore, macrophages exert a pro-inflammatory and pro-fibrotic role in the process of kidney fibrosis (16, 18). We found that the macrophage infiltration in obstructed kidneys was reduced significantly, which was beneficial for alleviating fibrosis.

β-OHB, which accounts for approximately 70% of the total ketone bodies, is produced by the liver and transported to the heart and kidney (19, 20, 30). As well as being an energy carrier, β-OHB has been certified to have anti-oxidative, anti-pyroptosis, and anti-inflammatory effects and associates with multitudinous signaling pathways, including the NLRP3 inflammasome, NF-κB, FOXO3, endoplasmic reticulum stress, histone deacetylases, and autophagy signaling pathways (20, 21, 31–35). Furthermore, β-OHB inhibits tumor growth and protects from ischemia-reperfusion injuries in the heart, kidney, and liver (34–37). We first observed that β-OHB restores Cpt1a and PPARα expression and, thus, mitigated fibrosis induced by TGFβ1 in TECs. PPARα, as a nuclear receptor transcription factor, participates in the regulation of FAO and oxidant production in mitochondria and peroxisomes (7, 8). PPARGC1a cooperates with PPARα, which modulates energy metabolism (7, 10). Proximal tubule PPARα and PPARGC1a attenuates renal fibrosis and inflammation induced by UUO and other injuries (38–41). In our study, KD and β-OHB significantly increased the expression of PPARα and PPARGC1a in obstructed kidneys and TECs, which activated the downstream genes of FAO. The network of interactions among AMPK, PPARα, and PPARGC1a, is involved in regulating cellular energy homeostasis. Most notably, AMPK stimulates mitochondrial biogenesis through the activation of PPARGC1a and alleviates the inhibition of Cpt1a (40, 42–44). Based on our results, β-OHB increases the phosphorylation of AMPK and indirectly enhances the expression of Cpt1a in TECs.

β-OHB binds to two G protein-coupled receptors, HCAR2 and FFAR3. HCAR2 (also known as Gpr109A) is expressed in various cell types such as immune cells, adipocytes, and colonic epithelial cells. Its activation inhibits adipocyte lipolysis and induces anti-inflammatory effects (19, 20, 45, 46). We observed that treatment with β-OHB did not change the expression of HCAR2 in TECs. FFAR3 (also known as Gpr41) is highly expressed in sympathetic ganglions. β-OHB antagonizes FFAR3 and suppresses sympathetic nervous system activity (47). However, another study reported that β-OHB, as an agonist for FFAR3, modulates sympathetic neurons (48). Whether agonism or antagonism of FFAR3 by β-OHB may depend on the specific tissue and disease contexts. Furthermore, FFAR3 signaling mediates glucose-stimulated insulin secretion and maintains energy homeostasis (49–51). In this study, the expression of FFAR3 was significantly decreased in TECs stimulated by TGFβ1, while β-OHB significantly enhanced its expression, suggesting that activation of FFAR3 may be controlled by β-OHB. We further confirmed that β-OHB functions via the FFAR3-dependent pathway by the agonist activating and siRNA blocking FFAR3.

Some limitations in our work should be noted. First, this study necessitates further exploration of the molecular mechanisms in knockout mice. Second, recent studies showed a more remarkable protective effect of KD than exogenous supplementation of ketone bodies, which suggests that KD is incompletely dependent on ketone bodies to fulfill its functions (5, 6). Accumulating evidence suggests that KD reduces intestinal pro-inflammatory Th17 cells and alleviates colitis by altering intestinal microbiota (52–54). Therefore, exogenous supplementation of β-OHB does not completely supersede KD, which induces more extensive systematic metabolic changes. Our work of β-OHB *in vitro* cannot fully elucidate the mechanisms of KD on renal fibrosis *in vivo*. Lastly, this study focused on HCAR2 and FFAR3 regulated by β-OHB. It has been reported that there are many G protein-coupled receptors, including HCAR1/3 and FFAR1/2, which regulate cellular and physiological functions in the body (55). Further research is required to understand how other receptors are involved in FAO.

## Conclusion

In conclusion, our current study elucidated the mechanism underlying the protective effect of KD on renal fibrosis through the enhancement of FAO and reduction in macrophage infiltration. These results shed new light on the role of energy metabolism disturbances in the progression of renal fibrosis and provide a potential therapeutic approach for CKD and renal fibrosis.

## Data availability statement

The datasets presented in this study can be found in online repositories. The names of the repository/repositories and accession number(s) can be found below: https://www.ncbi.nlm.nih.gov/genbank/, NM_001108912.1.

## Ethics statement

The animal study was reviewed and approved by the Institutional Animal Care and Use Committee of Tongji Medical College of Huazhong University of Science and Technology.

## Author contributions

YQ contributed to research design, performance of the research, data analysis, and the writing of the paper. XH contributed to the experimental design and assisted with data analysis. CX and CL participated in establishment of model and sample collection. RC and YX participated in data analysis and revised the article. JY conceived the study, designed the experiments, and supervised the research. All authors contributed to manuscript revision and read and approved the submitted version.
