# Supplementary material for: Ketogenic diet alleviates renal fibrosis in mice by enhancing fatty acid oxidation through the free fatty acid receptor 3 pathway
Source: Front Nutr. 2023 Mar 23;10:1127845. doi: 10.3389/fnut.2023.1127845 (PMC10081144; doi:10.3389/fnut.2023.1127845)
Supplement: Supplementary file 1 [file Table_1.DOCX]

Supplementary Material

Table S1 Primer sequences.

| Species | Gene | Forward sequence (5'→3') | Reverse sequence (5'→3') | |
| --- | --- | --- | --- | --- |
| Mouse | α-SMA | GTCCCAGACATCAGGGAGTAA | TCGGATACTTCAGCGTCAGGA | |
|  | Col1a1 | TAAGGGTCCCCAATGGTGAGA | GGGTCCCTCGACTCCTACAT | |
|  | Col3a1 | CTGTAACATGGAAACTGGGGAAA | CCATAGCTGAACTGAAAACCACC | |
|  | Fn-1 | ATGTGGACCCCTCCTGATAGT | GCCCAGTGATTTCAGCAAAGG |  |
|  | Cpt1a | TGGCATCATCACTGGTGTGTT | GTCTAGGGTCCGATTGATCTTTG | |
|  | Acox1 | TAACTTCCTCACTCGAAGCCA | AGTTCCATGACCCATCTCTGTC | |
|  | PPARα | AACATCGAGTGTCGAATATGTGG | CCGAATAGTTCGCCGAAAGAA | |
|  | PPARGC1a | TATGGAGTGACATAGAGTGTGCT | GTCGCTACACCACTTCAATCC | |
|  | CD36 | AGATGACGTGGCAAAGAACAG | CCTTGGCTAGATAACGAACTCTG | |
|  | IL-1β | GAAATGCCACCTTTTGACAGTG | TGGATGCTCTCATCAGGACAG | |
|  | IL-6 | CTGCAAGAGACTTCCATCCAG | AGTGGTATAGACAGGTCTGTTGG | |
|  | TNF-α | CAGGCGGTGCCTATGTCTC | CGATCACCCCGAAGTTCAGTAG | |
|  | GAPDH | TGACCTCAACTACATGGTCTACA | CTTCCCATTCTCGGCCTTG | |
| Rat | α-SMA | CAGCTATGTGGGGGACGAAG | TCCGTTAGCAAGGTCGGATG | |
|  | Col1a1 | GATCCTGCCGATGTCGCTAT | GGAGGTCTTGGTGGTTTTGTATTC | |
|  | Fn-1 | CCCCAACTGGTTACCCTTCC | TGGTTCGCCTAAAGCCATGT | |
|  | Vim | TGAGATCGCCACCTACAGGA | GGAGTGGGTGTCAACCAGAG | |
|  | Acox1 | GGGCACGGCTATTCTCAC | GACTCGGCAGGTCATTCA | |
|  | Cpt1a | CCTACCACGGCTGGATGTTT | TACAACATGGGCTTCCGACC | |
|  | PPARα | GAAGCCCAAATAGACAGC | AAACAGATTGACTCGCACT | |
|  | HCAR2 | CTCGACCCGGTGGTCTACTA | ATCTGGTTCGCCCAAGGTTT | |
|  | GAPDH | CGTATCGGACGCCTGGTT | CGTGGGTAGAGTCATACTGGAA | |

α-SMA: α-smooth muscle actin; Col1a1: collagen type I alpha 1 chain; Col3a1: collagen type III alpha 1 chain; Fn-1: fibronectin; Cpt1a: carnitine palmitoyltransferase 1a; Acox1: acyl-coenzyme A oxidase 1; PPARα: peroxisome proliferator-activated receptor-α; PPARGC1a: PPAR-γ coactivator-1a; CD36: cluster of differentiation 36; IL: interleukin; TNF-α: tumor necrosis factor-α; Vim: vimentin; HCAR2: hydroxycarboxylic acid receptor 2.
